# The role of ILC2s in asthma combined with atopic dermatitis: bridging the gap from research to clinical practice

**DOI:** 10.3389/fimmu.2025.1567817

**Published:** 2025-04-01

**Authors:** Yan-fang Luo, Yu Deng, Feng Yang, Meiduosiji, Xia Xiong, Yu-lai Yuan, Su-hua Ao

**Affiliations:** ^1^ Department of Respirology, The Affiliated Traditional Chinese Medicine Hospital, Southwest Medical University, Luzhou, Sichuan, China; ^2^ College of Integrated Chinese and Western Medicine, Southwest Medical University, Luzhou, Sichuan, China; ^3^ Department of Dermatology, The First Affiliated Hospital of Southwest Medical University, Luzhou, Sichuan, China

**Keywords:** group 2 innate lymphoid cells, asthma, atopic dermatitis, asthma combined with atopic dermatitis, biologics

## Abstract

Asthma, a complex and heterogeneous respiratory disease, is often accompanied by various comorbidities, notably atopic dermatitis (AD). AD characterized by recurrent eczematous lesions and severe itching, can trigger or exacerbate asthma. Individuals with AD are 2.16 times more likely to develop asthma compared to the reference population. Furthermore, asthmatics with AD experience more severe and frequent emergency department visits and hospital admissions compared to patients with asthma alone. The close connection between asthma and AD indicates there are overlap pathophysiologic mechanisms. It is well-known that dysregulated type 2 (T2) immune inflammation is pivotal in the development of both AD and asthma, traditionally attributed to CD4^+^ type 2 helper T (Th2) cells. Over the past decade, group 2 innate lymphoid cells (ILC2s), as potent innate immune cells, have been demonstrated to be the key drivers of T2 inflammation, playing a crucial role in the pathogenesis of both asthma and AD. ILC2s not only trigger T2 immune-inflammation but also coordinate the recruitment and activation of innate and adaptive immune cells, thereby intensifying the inflammatory response. They are rapidly activated by epithelium alarmins producing copious amounts of T2 cytokines such as interleukin (IL) -5 and IL-13 that mediate the airway inflammation, hyperresponsiveness, and cutaneous inflammation in asthma and AD, respectively. The promising efficiency of targeted ILC2s in asthma and AD has further proven their essential roles in the pathogenesis of both conditions. However, to the best of our knowledge, there is currently no review article specifically exploring the role of ILC2s in asthma combined with AD and their potential as future therapeutic targets. Hence, we hypothesize that ILC2s may play a role in the pathogenesis of asthma combined with AD, and targeting ILC2s could be a promising therapeutic approach for this complex condition in the future. In this review, we discuss recent insights in ILC2s biology, focus on the current knowledge of ILC2s in asthma, AD, particularly in asthma combined with AD, and suggest how this knowledge might be used for improved treatments of asthma combined with AD.

## 1 Introduction

Over the past decades, the incidence of allergic diseases, such as AD and bronchial asthma (asthma), has significantly increased, affecting approximately 20% of the world’s population, thereby imposing a substantial individual and socioeconomic burden (1, 2). Atopic diseases may evolve from one to another. Intriguingly, AD often serves as the first step indicating the development of other allergic diseases, such as food hypersensitivity, allergic rhinitis, and asthma, namely the “atopic march” (3). The comorbidity of asthma and AD has garnered significant research attention due to the strong association between AD and the development, severity, and risk of exacerbations in asthma (3–5). A comprehensive meta-analysis involving 458,810 participants indicated that individuals with AD were 2.16 (95% confidence interval [CI], 1.88-2.48) times more likely to develop asthma compared to those without AD (6). Recent research by Ahn et al. utilized a two-sample bidirectional Mendelian randomization approach to reinforce the causal link between AD and the subsequent development of asthma (7). And the risk for progression to asthma was correlated with the severity of AD that severe AD was significantly (relative risk [RR], 2.40; 95% CI, 1.96-2.94) higher than in mild AD (RR, 1.82; 95% CI, 1.03-3.23) or moderate AD (RR, 1.51; 95% CI, 1.30-1.75) (6). Moreover, asthma combined with AD has worse clinical outcomes compared with asthma only (8). In a population-based cohort study including 65,539 individuals with asthma only and 819 with concomitant asthma and AD, the latter had more emergency department visits (9% vs 7%) and hospital admissions (31% vs 27%) compared to patients with asthma only (8). These results suggested that improvements in management and monitoring AD may reduce unscheduled hospital visits and lower healthcare costs in asthmatics combined with AD. Therefore, McDonald et al. have suggested that AD was an important extrapulmonary treatable traits of asthma since it could be modifiable by targeted treatment to improve the outcomes of asthma (9).

The close connection between AD and asthma indicates that they have overlapping pathogenetic mechanisms. It is clearly that dysregulated T2 immune response is one of the fundamental causes of AD and asthma (10). Although CD4^+^ Th2 cells undoubtedly play an important role in the pathogenesis of AD and asthma, the discovery of ILC2s has added another layer of complexity to the pathogenesis of these diseases. Increasing evidence suggests that ILC2s as potent innate immune cells are key drivers of T2 inflammation (11). In a freshly released research, Szeto et al. applied the Boolean-ILC2-Cre mice to definitively establish the crucial role of ILC2s in facilitating the initiation of Th2-driven adaptive immunity (12). They are not only initiating T2 immune-inflammation but orchestrating the recruitment and activation of other members of innate and adaptive immunity, further amplifying the inflammatory response (13).

Despite their lower total numbers compared to Th2 cells in the body, ILC2s respond more swiftly and remain unaffected by antigen stimulation (14). ILC2s are rapidly activated by epithelium alarmins producing copious amounts of T2 cytokines such as IL-5 and IL-13 (14). The promising efficacy of targeting ILC2s in AD and asthma (14) has further proved their essential roles in the pathogenesis of AD and asthma. However, their mechanism of action in asthma combined with AD has not yet been completely elucidated. In this review, we explore recent advances in ILC2s biology, emphasizing their role in asthma and AD, especially in the context of asthma combined with AD, and how this knowledge can be leveraged to develop improved treatments for asthma combined with AD.

## 2 Immunobiology of ILC2s

For slightly over a decade, ILC2s, identified as one of the five key members of the innate lymphoid cell (ILC) family, play a crucial role in the body’s initial defense against infections (15). Alongside natural killer (NK) cells, ILC1s, ILC3s, and lymphoid tissue inducer cells (LTi), ILC2s contribute to the innate immune response that precedes the activation of adaptive immune cells (13). ILC2s are abundant in various mucosal tissues, including lung, skin, and intestine, where they exhibit tissue-specific heterogeneity (16). A recent single-cell RNA sequencing study revealed distinct patterns of receptor expression in tissue-specific ILC2 populations. Specifically, cutaneous ILC2s demonstrated low expression levels of IL-33 receptor (ST2), IL-25 receptor (IL17RB), and TSLP receptor (TSLPR), but exhibited marked upregulation of IL-18 receptor. In contrast, lung ILC2 populations showed reciprocal expression profiles, characterized by high ST2 expression coupled with minimal IL-18 receptor detection (17). We have summarized the different expression receptors of ILC2s in lung and skin (see **Table 1**) (17–19).

**Table 1:** Different receptors of ILC2s in lung and skin^17-19^.

	ST2	IL-17RB	TSLPR	IL-2Rα	IL-4Rα	IL-7Rα	IL-18R1	CRTH2	ICOS	IL-9Rα	CCR2	CCR6	Klrg1	Nrp1	HTR2A	IP	NMUR1	Itgae
Lung	+^hi^	+	+	+	+	+	+^lo^*	+^hi^	+	+	+	n.d.	+	+	+*	+*	+	n.d.
Skin	+^lo^	+^lo^	+	+	+	+	+^hi^	+^lo^#	+^hi^	n.d.	n.d.	+	n.d.	n.d.	n.d.	n.d.	n.d.	+*

+, expression; +^hi^, highly expressed; +^lo^, low expression; n.d., not determined; *, only expressed in mouse tissues; #, only expressed in human tissues. IL, interleukin; ST2, IL-1 receptor-like 1 (IL1RL1); IL-17RB, IL-17 receptor B; TSLPR, Thymic stromal lymphopoietin receptor; IL-2Rα, IL-2 receptor alpha; IL-4Rα, IL-4 receptor alpha; IL-7Rα, IL-7 receptor alpha; IL-18R1, IL-18 receptor 1; CRTH2, Chemoattractant receptor-homologous molecule expressed on T helper type 2 cells; ICOS, Inducible T cell costimulator; IL-9Rα, IL-9 receptor alpha; CCR2, C-C motif chemokine receptor 2; CCR6, C-C motif chemokine receptor 2; Klrg1, killer cell lectin-like receptor subfamily G member 1; Nrp1, Neuropilin-1; HTR2A, 5-hydroxytryptamine receptor 2A; IP, Prostaglandin I2 receptor; NMUR1, neuromedin-U receptor 1; Itgae, Integrin alpha E.

**Figure 1:**
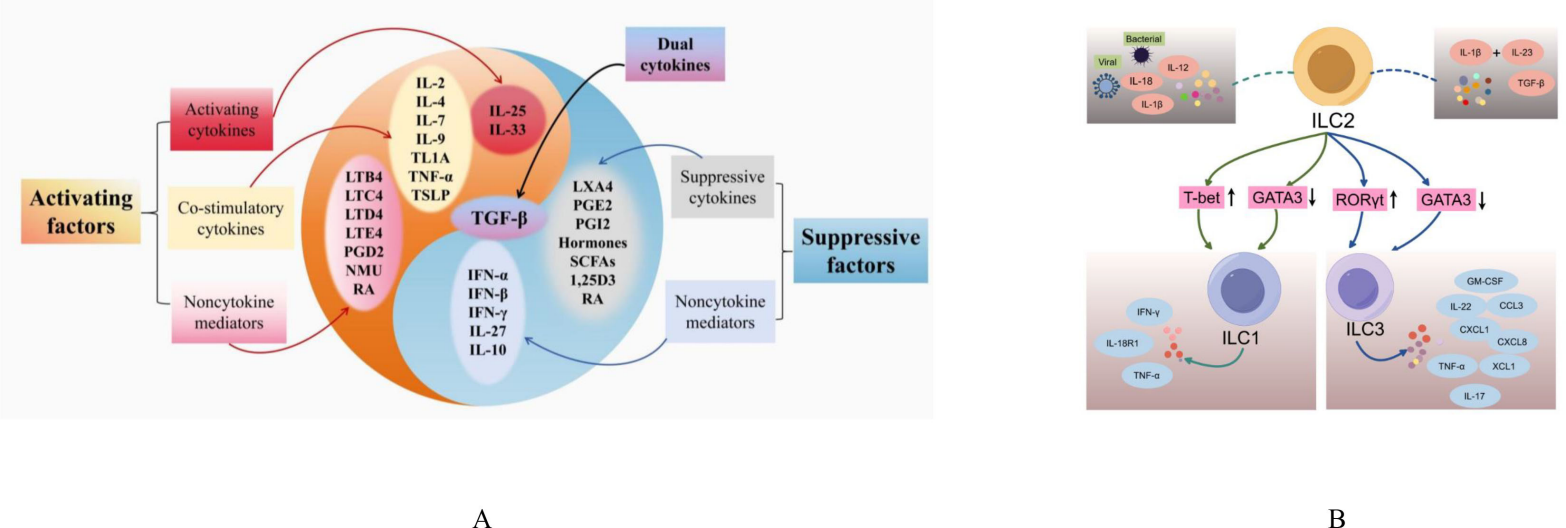
The modulating factors and differentiation of ILC2s. **(A)** Activating and suppressive factors of ILC2s; **(B)** ILC2s Plasticity. IL, interleukin; TSLP, thymic stromal lymphopoietin; IFN, interferon; TLA1, tumor necrosis factor-like cytokine 1A; TNF-α, tumor Necrosis Factor alpha; TGF-β, transforming growth factor beta; LT, leukotrienes; PG, prostaglandins; NMU, neuromedin U; RA, the vitamin A metabolite retinoic acid; LXA4, Lipoxin A4; 1,25D3, vitamin D metabolite1, 25-dihydroxyvitamin D3; SCFA, short-chain fatty acids; INF, Interferon; T-bet, T-box expressed in T cells; GATA3, GATA Binding Protein 3; RORγt, Retinoic Acid Receptor-related Orphan Receptor Gamma t; IL-18R1, IL-18 receptor 1; GM-CSF, Granulocyte-Macrophage Colony-Stimulating Factor; CCL3, Chemokine (C-C motif) Ligand 3; CXCL, Chemokine (C-X-C motif) Ligand; XCL1, X-C motif chemokine ligand 1.

Initially, they were considered to be associated with resistance to parasitic infections (15). Nowadays, it is increasingly clear that ILC2s play pivotal roles in the regulation of body’s homeostasis and tissue repair (20). Moreover, they can also regulate the function of other T2 immune cells such as Th2 cells which share key transcription factors and cytokine production profiles with ILC2s (13). Therefore, ILC2s are often considered to be the “mirror cell” of Th2 cells (21). Although their total cell numbers are far less than the numbers of Th2 cells in the body, ILC2s respond faster and are not affected by antigen stimulation (13). This allows ILC2s to be the first responders to allergens that enter the system, producing a large amounts of T2 cytokines including IL-5 and IL-13, driving eosinophilia (22). Dysregulation of ILC2s contributes to T2-skewed immune-mediated inflammatory diseases such as AD and asthma, making ILC2s to be an attractive target for therapeutic interventions (13). However, there are complex regulatory systems to modulate ILC2s functions and plasticity. The abnormal regulatory systems would result in ILC2s dysfunction that leads to unfavorable health problems. In the coming section, we focus on the regulatory systems and molecules that modulate ILC2s’ functions and plasticity (**Figure 1**).

### 2.1 Activating cytokines and co-stimulatory cytokines

Unlike T cells, ILC2s do not possess antigen-specific receptors, therefore, their activation is mediated by the recognition of epithelial-derived cytokines (alarmins), mainly, IL-25 and IL-33 (22). They are strong activators of ILC2s via mitogen-activated protein kinases (MAPKs) and tumor necrosis factor receptor (TNFR)-associated factor 6 (TRAF6) pathways, respectively (23–26). IL-25 belongs to IL-17 family cytokines which is released from epithelium, including tuft cells and brush cells (27). In mouse models, IL-25 can amplify the T2 immune response, significantly induce the expression of the IL-4/5/13 cytokines gene, and causes lesions in the lung and digestive tract (27). And the administration of IL-25 has been shown to induce lung eosinophilia and airway hyperreactivity (AHR), underscoring its critical role in the pathogenesis of asthma (27). IL-33 belongs to the IL-1 family and is released from epithelial cells, fibroblasts, endothelial cells, among others (28). It elicits the activation of ILC2s and stimulates ILC2s to produce IL-5 and IL-13 in mouse and human (29). In humans, endothelial cells and bronchial epithelial cells constitutively express IL-33 and may serve as major sources during airway inflammation (29).

However, mouse and human studies indicate that the application of IL-25 or IL-33 alone has limited effects on activating ILC2s, necessitating additional stimulus signals (30, 31). Common co-stimulators are categorized into two primary groups: the gamma-C (γ-c) cytokine family, comprising IL-2 and IL-15 (32), and the tumor necrosis factor superfamily (TNFSF), which encompasses a range of ligands and receptors involved in various immune and inflammatory processes (33). The γ-c cytokine family, which encompasses IL-2, IL-4, IL-7, IL-9, and others, activates ILC2s through the Janus kinase (JAK)/signal transducer and activator of transcription (STAT) pathway (32). These cytokines play a crucial role in the survival, development, and maintenance of ILC2s (32). Mouse studies found that IL-2 and IL-7 can strongly activate STAT5 pathway, and collaborative IL-33-induced ILC2s proliferation and producing T2 cytokines (34–36). Similar to IL-2 and IL-7, IL-4 and IL-9 can amplify cytokines production by activated mouse and human ILC2s in synergy with IL-33 (36, 37). Motomura et al. found that basophil-derived IL-4 facilitated ILC2s effector functions in papain-induced mouse airway inflammation (38). While autocrine IL-9 induces the anti-apoptotic protein to support mouse ILC2s survival (39).

Additionally, the current body of evidence has shown that members of TNFSF possess costimulatory functions, particularly tumor necrosis factor-like cytokine 1A (TL1A) and tumor necrosis factor-α (TNF-α) (40, 41). TL1A activates ILC2s through its cognate receptor DR3 expressed on mouse and human ILC2s, leading to activation of the nuclear factor kappa-B (NF-кB) and MAPK signaling pathways (42). In synergy with IL-33 and IL-25, TL1A can enhance mice ILC2s functions, mediate papain-induced ILC2s activation and lung inflammation (43, 44). TNF-α binds to its receptor TNFR2 which is expressed on mouse ILC2s, promoting ILC2s activation and survival through the non-canonical NF-кB pathway (41). Furthermore, the IL-33 and A. alternata-challenged asthma mouse model has demonstrated that TNF-α/TNFR2 signaling is crucial for ILC2s survival and function and induction of AHR (41).

Thymic stromal lymphopoietin (TSLP) as one of the alarmins is historically considered to be an activator of ILC2s, but increasing evidence suggest that this factor has costimulatory properties similar to IL-2 and IL-7 (26). TSLP alone does not induce mouse ILC2s to produce cytokines, it acts in synergy with IL-33 to enhance cell viability, proliferation, and cytokine production (36). Moreover, TSLP augments IL-33-induced IL-9 production which can amplify the cytokines production of mouse ILC2s (36). Intriguingly, TSLP is more important for ILC2s survival than IL-33 in humans and plays a critical role in ILC2s mediated steroids resistance (45).

### 2.2 Suppressive cytokines

Mouse and human ILC2s’ functions can be attenuated by a spectrum of cytokines, such as interferons (IFNs), IL-27, IL-10, and transforming growth factor-β (TGF-β) (26). *In vivo* and *in vitro* studies have demonstrated that IFNs and IL-27 can strongly suppress the proliferation of ILC2s and production of type 2 cytokines by ILC2s through STAT1 pathway (46). Exogenous administration of IFNs (IFN-β or IFN-γ) can inhibit mouse and human ILC2s’ effector functions and relieve the flowing T2 immunopathology (46, 47). IL-27 is another cytokine that directly inhibits mouse ILC2s’ functions in a manner dependent on the STAT1 (46). In the A. alternata-induced asthmatic animal model, administration of IL-27 was not only significantly suppress the number of ILC2s cells in bronchoalveolar lavage fluid (BALF) and lung, but also attenuate ILC2-driven airway inflammation (48).

IL-10 inhibits mouse ILC2s proliferation and T2 cytokines production in response to IL-33/TSLP stimulation (49). In addition, a recent clinical research suggested that IL-10-producing ILC2s attenuate Th responses and maintain epithelial integrity in modulating grass pollen allergy (50). The role of IL-10^+^ ILC2s in the disease-regulatory role of allergen immunity is highlighted (50). However, IL-10 failed to inhibit IL-25-activated mouse ILC2s, indicating that its inhibition of ILC2s depends on the stimulator (51, 52). TGF-β is an important cytokine for ILC2s development and maturation, as TGFBR2-deficient mice exhibit reduced numbers of mature ILC2s in various tissues, including the lungs (53). Moreover, a clinical study has shown that TGF-β not only inhibits the production of IL-5 and IL-13 by ILC2s but also increases the production of IL-9 which acts as a costimulator of ILC2s (49). Therefore, compared with other inhibitory cytokines, TGF-β plays a dual role in the activation of ILC2s (54). Further studies are needed to clarify the role of TGF-β in ILC2s’ functions.

### 2.3 Noncytokine mediators

In addition to cellular mediators, ILC2s are also regulated by non-cytokine mediators, such as lipid mediators, neuropeptides/neurotransmitters, hormones, among others. It is well known that lipid mediators including leukotrienes (LT) and prostaglandins (PG) play crucial roles in homeostasis and inflammation (55). Both human and mouse ILC2s possess a variety of receptors for LT and PG. LTB4, LTC4, LTD4, LTE4, and PGD2 are known to positively regulate ILC2s (56–60). Meanwhile, LXA4, PGE2, and PGI2 are negative regulators of ILC2s (61–63). Neuropeptides produced by peripheral neurons have been suggested could directly affect the function of ILC2s in the lung and intestine (64). As a strong positive regulator, neuromedin U (NMU) rapidly activates human ILC2s through the NMU/NMUR1 axis (65). In addition, the abundance of the neurotransmitter dopamine is negatively correlated with the amount of circulating mouse and human ILC2s, which alleviates allergen-induced ILC2s responses and airway inflammation by impairing the mitochondrial oxidative phosphorylation (OXPHOS) pathway of ILC2s (66). In turn to hormones, more and more studies indicate that sex hormone plays an important role in the regulation of ILC2s (67–70). Testosterone and its downstream hormone, 5α-dihydrotestosterone, negatively regulate the proliferation of mouse ILC2s and the production of type 2 cytokines by down-regulating the expression of GATA binding protein 3 (GATA3) and Retinoic Acid-Related Orphan Receptor Alpha (RORα) (69). On the other hand, while estrogen does not affect the lung ILC2s signal, a lack of mice uterine ILC2s reduces estrogen receptors, estrogen-related to maintaining permanent ILC2s uterus (70).

Furthermore, vitamin metabolites and dietary metabolites have demonstrated the ability to regulate ILC2s function, either directly or indirectly (26). The vitamin A metabolite retinoic acid (RA) acts different functions in ILC2s of different species; in humans, RA induces ILC2s to produce T2 cytokines (71), whereas in mice, RA deficiency promotes ILC2s proliferation and cytokine production (72). In addition, the vitamin D metabolite, 1,25-dihydroxyvitamin D3, exerts an inhibitory effect on human ILC2s (71). It is well-established that diet plays a crucial role in influencing immune homeostasis and the development of allergic diseases (73). Animal research has found that short-chain fatty acids (SCFAs) mainly produced by dietary fiber can reduce ILC2-mediated allergic inflammation (74). Recent research has demonstrated that butyrate, a type of SCFAs, can suppress the production of type 2 cytokines and proliferation of ILC2s by targeting GATA3 in both mice and humans (75). Meanwhile, inhibiting the mouse ILC2s’ functions through the administration of butyrate significantly improved the AHR and airway inflammation mediated by A. alternata and IL-33 (76). Interestingly, programmed cell death protein-1 (PD-1) as a potent immune inhibitory receptor is well-known in tumor therapy (77), which is also expressed on mouse ILC2s and acts an early checkpoint in ILC2s (78, 79). Recently, PD-1 was reported to limit the viability of ILC2s and attenuated their effector functions by shifting mouse ILC2s metabolism toward glutaminolysis, glycolysis and methionine catabolism (80).

### 2.4 ILC2s plasticity

For some time, ILC2s were considered to be a homogeneous population of cells because, no phenotypic or functional heterogeneity of ILC2s were found like ILC1s and ILC3s (13). Nowadays, it is clear that ILC2s exhibit high plasticity, as evidenced by their capacity to differentiate into cells with characteristics of ILC1 or ILC3 subsets in response to microenvironmental signals (81). Animal study has shown that IL-1β and IL-12 produced by viral and bacterial infection can drive ILC2s converts to IFN-γ-producing ILC1-like cells which was associated with disease severity and acute exacerbation of chronic obstructive pulmonary disease (82). Moreover, recent animal research has shown that stimulating ILC2s with a mix of IL-1β, IL-23, and TGF-β leads to the upregulation of RORγt, a key transcription factor for ILC3s, while concurrently downregulating GATA3 and inducing IL-17 production (83). As IL-17 is significantly associated with airway neutrophilia, the transdifferentiation of human ILC2s into ILC3-like cells might contribute to neutrophilic asthma (84). Thus, the unique high plasticity of ILC2s suggests that they may play an important role in multiple immune responses against different types of pathogens/infections (83).

In conclusion, studies examining the stimulators, suppressors, plasticity, and other factors associated with ILC2s have furnished invaluable insights into the understanding of ILC2s and their potential exploitation for developing novel therapeutic strategies to combat allergic diseases.

## 3 Asthma and ILC2s

Asthma is a very common and heterogeneity chronic respiratory disease characterized by chronic airway inflammation and AHR, affecting more than 300 million people worldwide across all age ranges (85). With the increased understanding of its heterogeneity, many clinical phenotypes of asthma have been identified, such as allergic asthma, non-allergic asthma, late-onset asthma, early-onset asthma, and asthma associated with obesity (2). In terms of causes, manifestations, and treatment response, phenotypes are clinically relevant, however, they do not certainly associate with the underlying disease mechanisms (86). More than two decades ago, Wenzel et al. stratified severe asthmatics into two subtypes according to the presence of airway eosinophilia which might allow for more precise treatment (87). In 2008, Anderson introduced the concept of endotype in asthma, which refers to a functionally and pathologically defined subtype of the disease, characterized by specific molecular features (88). Subsequently, Lötvall and colleagues defined an endotype as “a subtype of a condition, which is defined by a distinct functional or pathophysiological mechanism.” (89). Endotype is considered to be a subtype defined by an unique pathophysiological mechanism at the cellular and molecular level which link molecular mechanisms to phenotype (90). As research deepens, asthma has subsequently been polarized into two major endotypes: T2-high and T2-low, which represent the most established classification, particularly in considering biologic therapy (91). These endotypes are defined based on their level of expression of T2 cytokines, such as IL-4, IL-5, and IL-13, which may be secreted by Th2 cells or ILC2s, thus the terminology of subtype shifts from Th2 to T2 (90–93).

ILC2s are essential for eosinophils and T2 inflammation. Since the discovery of ILC2s, they have been shown to mediate airway inflammation and AHR in several asthmatic mouse models (80). And increasing data correlate ILC2s numbers and function with asthma incident and severity in humans (80). Allergens such as house dust mite (HDM) and ovalbumin (OVA) can activate mice ILC2s within a few hours, leading to the production of significant amounts of IL-13 and IL-5 (93–96). This immune response is associated with the development of asthma-like symptoms in mice, characterized by eosinophil infiltration, AHR, and mucus hypersecretion (57, 94–97). In addition to allergens, respiratory viral infections have been reported to enhance ILC2s activation and drive AHR and airway inflammation in mouse models, which demonstrate ILC2s may play an important role in viral-induced asthma exacerbation (98). ILC2-deficient mice, however, had reduced eosinophil numbers and were unable to induce T2 inflammation at homeostasis (99). Recently, Jarick and colleagues used selective and specific ILC2-deficient mice to demonstrate the important role of ILC2s in airway inflammation in allergic asthma models (99). Moreover, ILC2s not only directly induce airway inflammation, but also activate Th2 cell responses through T2 cytokines released by ILC2s as signaling molecules. Particularly, mouse ILC2-derived IL-13 can induce dendritic cells to potentiate the memory Th2 cell response during allergen rechallenge (100). That is, ILC2s enhance T2 inflammation by orchestrating innate and adaptive T2 immunity.

It is clear that sex hormone plays an important role in the pathogenesis of asthma may also associated with ILC2s. Wang et al. have found female mice exhibited more ILC2s and T2 cytokines than male mice in a Rag1^-/-^ asthmatic model (101). *In vitro* study, treatment of testosterone significantly attenuated the production of ILC2s-derived T2 cytokines and the proliferation of ILC2s in response to IL-25/33 (101). Dopamine has been shown to inhibit mouse and human ILC2s responses by restraining mitochondrial activity (66). Conditional deletion of the ILC2s’ dopamine receptor Drd1 or ablation of lung dopaminergic neurons resulted in excessive ILC2s responses and aggravated airway inflammation in clinical asthma model (66). In recent years, an increasing number of researchers have recognized the crucial roles played by the PD-1/PD-L1 or PD-L2 signaling pathway in modulating inflammatory responses in asthma (66, 80, 102–104). Helou et al. have demonstrated that PD-1 deficiency functions as a metabolic checkpoint in mice ILC2s, influencing their activation and proliferation (80). And PD-1 agonist could ameliorate AHR and suppress lung inflammation in a asthmatic mouse model (80). More importantly, ILC2s were suggested one of the major sources of steroid-resistant IL-4 and IL-13 transcripts in mice models of steroid-resistant asthma exacerbation (105).

In line with animal studies, a growing number of clinical studies have demonstrated the close relationship among ILC2s and asthma (106, 107). Asthmatic patients exhibit significantly elevated levels of ILC2s in their peripheral blood, sputum, and BALF, in contrast to control subjects (108–110). Smith et al. and other researchers have reported that the frequency of ILC2s was positively correlated with disease severity and airway eosinophilic inflammation (sputum and blood eosinophils and fractional exhaled nitric oxide [FeNO] fraction) (111) and negatively correlated with predicted forced expiratory volume in 1 second (FEV1%) (108). Recently, human ILC2s have been reported in mediating resistance to immunosuppression by corticosteroids, a cornerstone drug for asthma (45, 112, 113), however, partial patients fail to response adequately (114, 115).

Considering the critical role of ILC2s in the pathogenesis of asthma, targeting ILC2s emerges as a promising therapeutic strategy for asthma, aligning well with the concept of precision medicine. Tiotropium as an effective long-acting muscarinic antagonist is approved for maintenance treatment of asthma in recent years (116). Adding tiotropium to high-dose inhaled corticosteroids plus long-acting Beta2-agonists has been shown to significantly improve lung function and reduce the risk of exacerbation in patients with moderate to severe asthma (117–120). Matsuyama et al. found tiotropium regulated mouse airway inflammation through ILC2s (121). It attenuated ILC2-dependent airway inflammation by suppressing basophils-derived IL-4 production and modulating ILC2s activation in a papain-induced asthma murine model (121). Moreover, some biological agents targeting ILC2s activators and co-stimulators have demonstrated efficacy in improving airway inflammation, AHR, and asthma symptoms in both animal experiments and clinical trials (122).

Mepolizumab, a humanized monoclonal antibody specifically targeting IL-5, has gained global approval as an add-on therapy for severe asthma patients who exhibit an eosinophilic phenotype (123). Across numerous clinical trials and real-world studies, the efficacy of mepolizumab in providing therapeutic benefits to these patients has been consistently demonstrated (124–129). Intriguingly, severe asthmatics treated with mepolizumab exhibited reduced ILC2s proliferation, TSLPR, GATA3 expression, and T2 cytokine secretion, accompanied by clinical improvement in asthma symptoms and a reduction in exacerbation frequency (107). The study suggests that post-treatment reduction in airway inflammation correlates with a diminished infiltration of ILC2s into the lungs (107). Dupilumab is a fully human monoclonal antibody against IL-4Rα, the receptor to IL-4 and IL-13, which was licensed for the treatment of asthma in 2018 (130). Numerous clinical trials have demonstrated significant improvements in asthma control and lung function following dupilumab treatment, resulting in a notable reduction in exacerbation rates (131–134). For instance, in the VENTURE study, dupilumab treatment led to a 70% reduction in the average dose of oral corticosteroids and a 59% reduction in asthma exacerbations among the overall patient population (134). In a murine model indicated IL-4 might facilitate ILC2s cytokine production through IL-4R (38). Dupilumab treatment significantly reduced ILC2s numbers in the blood of asthmatics and inhibited the expression of IL-5 and IL-13 mRNA in ILC2s (135). Thus, these results suggested that dupilumab attenuated ILC2s response in asthmatics and might be involved in the reduced risk of asthma exacerbation (135). Moreover, human ILC2s have been demonstrated to express IL-4Rα that means dupilumab can be a novel treatment targeting ILC2s (37). Tezepelumab, the first biologic targeting TSLP, was approved by the FDA in 2021 for the add-on maintenance treatment of severe asthma, regardless of phenotype or endotype (136). It has been shown to reduce exacerbations, improve lung function, and quality of life in patients with severe asthma (137, 138). As mentioned above, IL-33 and IL1RL1 (encoding ST2) have obvious links to ILC2s biology. The efficiency of biological agents targeting IL-33 (itepekimab) and its receptor (astegolimab) have been confirmed in clinical trials. In a phase 2 clinical trial involving 296 patients with moderate-to-severe asthma, itepekimab demonstrated significant improvements in asthma control, quality of life, and lung function, along with reductions in eosinophil levels and FeNO (139). Astegolimab has demonstrated the potential to reduce the annualized asthma exacerbation rate by up to 43% (140).Anti-IL-25 antibody significantly reduced serum IgE, IL-5, and IL-13 production, eosinophil infiltration (141); abrogated airway smooth muscle hyperplasia; prevented AHR in OVA and HDM induced asthma mouse models (142). Moreover, administration of anti-IL-25 neutralizing antibody attenuated ILC2s proliferation, mucous hypersecretion and AHR in rhinovirus infected mice model (143). SM17 (a humanized antibody of the IgG4/kappa isotype) targeting the IL-25 pathway via specific binding to IL-17RB, a co-receptor for IL-25 expressed on the surface of mouse and human ILC2s cells an other cells (144). The latest research has suggested that SM17 could significantly suppress T2 inflammation in the BALF and infiltration of ILC2s into the lungs in a HDM-induced asthma mouse model (145). Meanwhile SM17 was well tolerated with no drug-related serious adverse events were observed in the Phase I clinical study (145). These results indicates SM17 is a potential therapeutic agent to asthma with a favorable safety profile, and is worthy to further clinical development. Moreover, another anti-IL-25 monoclonal antibody, XKH001, has been evaluated as therapeutic agents in clinical trials for asthma (146).

## 4 ILC2s and atopic dermatitis

AD is an extremely common relapsing skin disorder characterized by recurrent eczematous lesions and intense itching, affecting individuals of all ages and ethnicities with at least 230 million patients worldwide (1). Moreover, AD is the leading cause of the global socioeconomic burden from skin disease (1, 147). The pathophysiology of AD is complex and multifactorial including genetic mutations, epidermal barrier dysfunction, skin microbiome abnormalities, and immune dysfunction (1). These pathologic drivers can interact with others (148). For example, the mutations of filaggrin gene (FLG) can induce the barrier dysfunction of skin which promotes inflammation and T2-cell infiltration; and local T2 immune responses further attenuate barrier function, and promotes dysbiosis (149). Among the multiple dysregulated immune responses in AD, T2-skewed immune dysregulation, driven by ILC2s and Th2 cells appears to be a dominant mechanism (150). In this section, we will focus on the roles of ILC2s in AD which mediated the skin inflammation by producing T2 cytokines such as IL-5 and IL-13.

ILC2s were found enriched in the skin lesions of AD patients and mice models, particularly in AD patients with FLG mutations and FLG-deficient mice (151, 152). Compared to healthy skin, the frequency of mouse and human ILC2s were significant increased in lesional AD skin, and the increase of IL-5 and IL-13 levels in the lesion area of AD were positively correlated with the number of ILC2s (153–155). Depletion of ILC2s attenuates AD-like dermatitis in mice, reinforcing the role of ILC2s in the development of AD (155). IL-33 as one of the most important alarmins to activate ILC2s is highly expressed in the skin of patients with AD (156). Excessive IL-33 has been demonstrated to cause AD-like inflammation in hK14mIL-33tg mice (transgenic mice for overexpress IL-33 in their skin) by producing large amounts of T2 cytokines, such as IL-4, IL-5,and IL-13, with increasing and activating ILC2s in the skin, lymph nodes, and peripheral blood (157). This was ILC2s-dependent for that hK14mIL-33tg mice with ILC2s deletion completely suppressed dermatitis and normalized the increasing of T2 cytokines (158). IL-25 as another important alarmins to activate ILC2s has been suggested to play an essential role in driving IL-13 expression by skin ILC2s to mediate skin inflammation in mouse AD model, both in acute and chronic phase (159). In terms of TSLP which has emerged as a key epithelium-derived cytokine and activator of skin ILC2s in the AD pathogenesis (157, 160–163). Increased expression of TSLP has been revealed in skin and serum from patients with AD (163, 164). Increasing evidence suggests that the PD-1 (can expressed on ILC2s and and acts an early checkpoint in ILC2s) and its ligands axis are emerging as key regulators of the immune response in allergic diseases including AD (165). The deficiency of PD-L1 leads to severe changes in the thickness of the ears and inflammation in AD murine model that indicates the involvement of PD-L1 in the pathogenesis of this disease (166). However, to date, there are no reports on the use of PD-L1 agonists to treat AD patients.

Given the important role of ILC2s in AD suggests that targeting ILC2s is a promising treatment of AD. Dupilumab as mentioned above is a fully human monoclonal antibody against IL-4Rα, which was the first biologic therapy to be approved for the treatment of moderate-to-severe AD in adults (167). In a clinical study in Japan, 27 AD patients treated with dupilumab had a significant reduction in the number of ILC2s in peripheral blood and total serum IgE, with significantly improvement the patient’s dermatitis (168). As mentioned above, SM17 as a humanized antibody of the IgG4/kappa isotype targets the IL-25 pathway, it could suppress T2 inflammatory response and ameliorate the AD pathology *in vitro* and *in vivo* data (143). Etokimab (ANB020), a humanized anti-IL-33 monoclonal antibody, reduced the peripheral eosinophils and skin neutrophil infiltration of AD patients in a proof-of-concept clinical trial (169). However, itepekimab (another anti-IL-33 monoclonal antibody) and astegolimab (anti-IL-33R monoclonal antibody), have recently shown not result in meaningful improvements in eczema area and severity index (EASI) scores versus placebo in a phase II clinical trial for moderate-severe AD patients (170). Recently, anti-TSLPR (ASP7266), a novel antibody, has been found to inhibit ILC2s activation, which almost completely inhibits antigen-specific cutaneous allergic reaction in the allergic experiment of AD monkey model (171). Similarly, inhibition of TSLP production by topical application of celastrol also resulted in the downregulation of ILC2s activation and alleviation of AD symptoms both *in vivo* and *in vitro* AD models (172). Tezepelumab (anti-TSLP) was investigated in a phase IIa trial in combination with topical corticosteroids (TCS) in adults with moderate to severe AD for 12 weeks (173). A higher number of patients in tezepelumab plus TCS-treated achieved response rate for ≥50% reduction in the EASI versus placebo plus TCS (64.7% vs 48.2% in the placebo arm; *P* = 0.091) (173), however, these improvements were not statistically significant. Although the results not statistically significant, numerical improvements versus placebo were found (173). Given the preclinical studies demonstrate the key role for IL-33 and TSLP in AD, additional clinical studies including more severe AD patients are required to validate these findings.

## 5 ILC2s in asthma combined with AD

AD is frequently present in patients with asthma, shares a strong genetic etiology with asthma, and is believed to be an important early causal factor in the ultimate development of atopic asthma (“atopic march”) (7, 174, 175). Li et al. conducted a meta-analysis with 458,810 participants to explore the link between AD and the progression to asthma (6). The results indicated that AD may be a precursor to asthma, as suggested by the “atopic march” concept (6). And individuals with AD were more than twice as likely to have asthma compared to the reference population (6). Recently, the casual relationship between AD and asthma has been confirmed by a two-sample bidirectional Mendelian randomization study (7). Furthermore, the risk of progressing to asthma was found to be correlated with the severity of AD (6). Amat et al. identified several risk factors for the development and progression of asthma in individuals with AD, including early-onset and severe AD, male gender, a parental history of asthma, and early and multiple sensitization (5). In addition, a population-based cohort study involving 65,539 individuals with asthma only and 819 with both asthma and AD found that patients with both conditions had a higher rate of emergency department visits (9% vs 7%) and hospital admissions (31% vs 27%) compared to those with asthma alone (8). These findings underscore the importance of AD as a significant comorbidity in severe asthma. Researchers emphasized that improved management and monitoring of AD could potentially reduce unscheduled hospital visits and lower healthcare costs (8). More recently, McDonald and colleagues highlighted AD as a crucial extrapulmonary treatable trait of asthma, noting that targeted treatments for AD could modify the condition and improve asthma outcomes (176).

**Figure 2:**
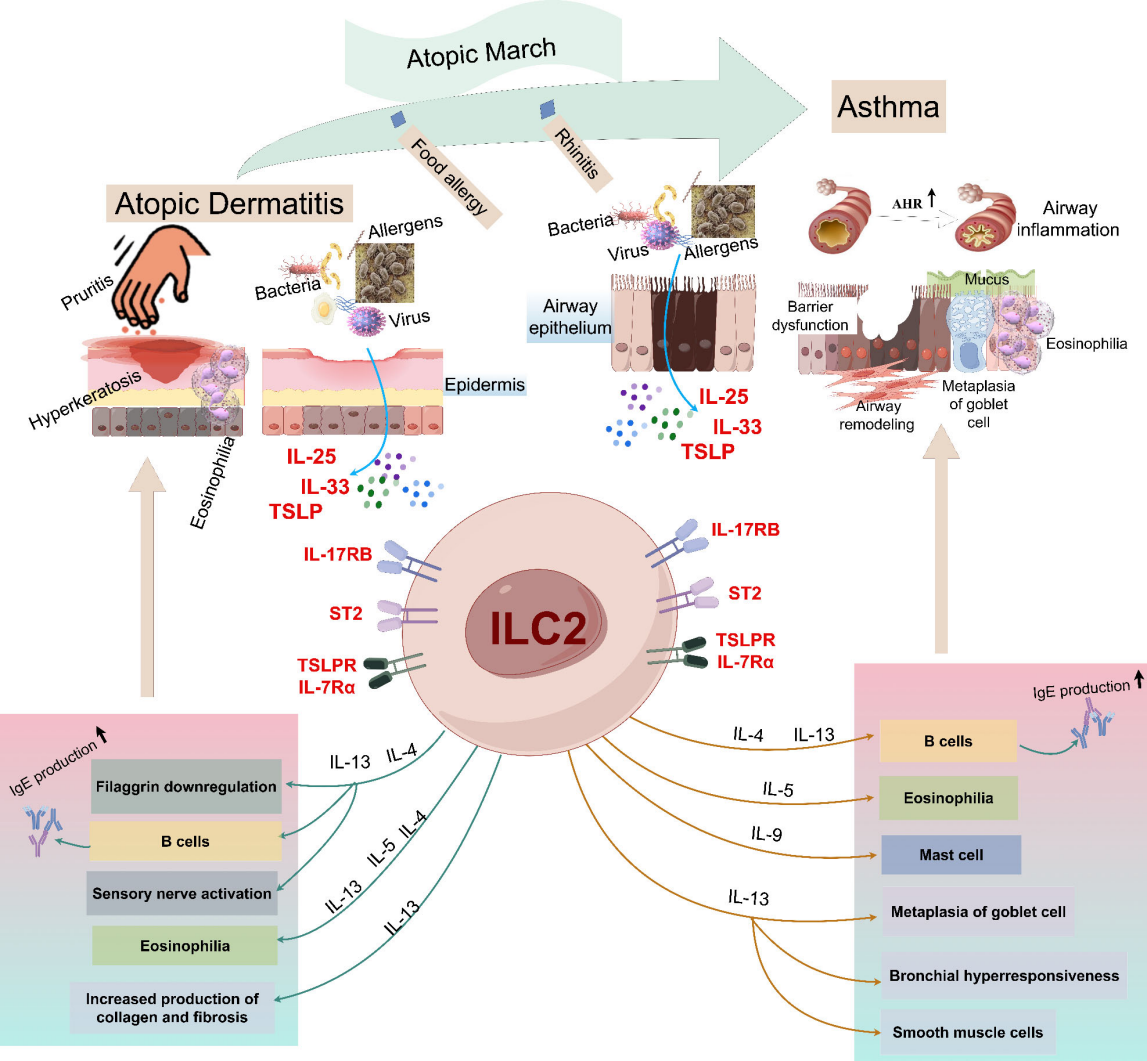
An illustrative overview of the roles of ILC2s in the pathogenesis of atopic dermatitis and asthma. Allergens enter the skin through the damaged skin barrier, which stimulates epithelial cells to release IL-25, IL-33 and TSLP, and then activates ILC2s, including IL-4, IL-5 and IL-13. These mediators cause the production of IgG1 and IgE, causing itching, skin inflammation and the decrease of skin barrier integrity, thus driving the onset of AD. When the allergen is encountered again, the alarm factors IL-25 and IL-33 released by the injured epithelial cells activate ILC2s, releasing various effector factors (including IL-4, IL-5, IL-9 and IL-13), among which IL-5 induces eosinophilia, IL-9 promotes mast cell growth and goblet cell metaplasia, and IL-4 and IL-13 pass through. IgG, Immunoglobulin G; IL, interleukin; ILC2s, group 2 innate lymphoid cells; AHR, airway hyperreactivity; ST2, IL-1 receptor-like 1 (IL1RL1); TSLP, thymic stromal lymphopoietin.

The close link between AD and asthma suggests that they share overlapping pathogenic mechanisms. Notably, dysregulated T2 immune inflammation mediated by ILC2s plays a pivotal role in the pathogenesis of both conditions, even in atopic march (**Figure 2**). However, the precise role of ILC2s in asthma combined with AD remains incompletely understood. In this section, we will delve into the current understanding of ILC2s in the context of asthma combined with AD, and explore potential implications for improved treatment strategies. By understanding the mechanisms underlying the role of ILC2s in this complex interplay, we may identify novel therapeutic targets that could benefit patients with asthma combined with AD.

In animal models, sensitization with allergens such as OVA, cockroach extract, or HDM followed by subsequent challenge successfully induced AD-like skin manifestations as well as airway inflammation and hyperresponsiveness (93, 153, 177–179). These findings provided evidence in support of the “outside-in” hypothesis, which posits that exposure to environmental allergens initiates the development of atopic conditions (177, 178). *In vivo* and *in vitro* studies have suggested that ILC2s might responsible for airway sensitization after skin exposure to allergens (179–182). Allergens through damaged skin barrier into the skin, stimulate epithelial cells to release alarmins including IL-25, IL-33 and TLSP. These cytokines activate ILC2s producing large amounts of T2 cytokines predominately IL-5 and IL-13, leading to AD when meeting again allergens, and can promote the occurrence of asthma independent of adaptive immunity (179). IL-5 is mainly responsible for the maturation and recruitment of eosinophils in the skin and airway (179). IL-13 damage the epithelial tight barrier and induce barrier leakiness in the skin and airway (180–182). Skin barrier injury sets into motion inflammation if these processes are continuing and the further generation of pro-inflammatory cytokines to establish a vicious cycle (10).

It is clearly that the epithelial and epidermal derived alarmins IL-25, IL-33, and TSLP play crucial roles in the action of allergic inflammation in asthma and AD. *In vitro* study, IL-25 acts synergistically with T2 cytokines to inhibit filaggrin expression and aggravate skin inflammation (183). And the expression of IL-25 was positively associated with airway inflammation and AHR in mouse study (184). IL-33 is highly expressed in skin or airway epithelial cells of AD or asthma animal models (185–188). IL-33 binds to IL-1 receptor appendages on a variety of cells through long-form serum stimulating factor-2 (ST2) and activates mouse and human ILC2s, leading to the development and aggravation of asthma and AD (188, 189). As a potent activator of ILC2s, TSLP is a key molecule in the progression of AD to asthma (190–192). Zhang and colleagues found that the increased expression of TSLP in skin keratinocytes not only triggered AD but also increased irritability asthma-like airway inflammation in a “atopic march” mice model by using calcipotriol topical, followed by OVA sensitization in the abdominal cavity and the nasal excitation (190). The results indicated that keratinocytic TSLP may act as an important mediator in the link of AD to asthma (190). Similarly, Jiang et al. established a mice model using topically calcipotriol, following by inhalation of HDM to induce asthma (193). It demonstrated that excessive production of TSLP in AD skin promoted airway sensitization, thereby triggered allergic asthma (193). The results indicated that TSLP represented a skin-transmitted signal in AD that promoted sensitivity to inhaled aeroallergens (193).

Given the critical role of ILC2s in asthma and AD, theoretically, biologics are effective by inhibiting ILC2s activation and proliferation in asthmatics combined with AD (**Table 2**). Excitingly, dupilumab as a human IgG4к monoclonal antibody blocking IL-4 receptor (a shared receptor for IL-4 and IL-13) is the first biologic approved both for AD (FDA in 2017 and EMA in 2019) and asthmatic patients (USA and Europe in 2018) (196). Subsequently, the significant efficiency of dupilumab for asthma combined with AD had been found in many case reports (197–199), and demonstrated by clinical trails (200) and real-world study (201). Boguniewicz et al. evaluated the efficiency of dupilumab for asthma combined with severe AD in four randomized, double-blinded, placebo-controlled trials (200). The results showed statistically and clinically significant improvement in asthma control Questionnaire-5 score and AD-related outcomes (EASI, peak pruritus numerical rating scale, and dermatology life quality index) with acceptable safety (200). In a latest retrospective study, the researchers used dupilumab to treat severe AD patients with asthma, a reduction in the number of ILC2s could improve the two diseases at the same time has a good curative effect (202). This may suggest that we can reduce the number of ILC2s by blocking the effector of ILC2s in the treatment of AD-asthma, thereby improving the disease. These results put in evidence that dupilumab should be give priority to asthmatics combined with AD. As mentioned above, tezepelumab as a human monoclonal antibody that inhibits the action of TSLP has licensed as an add-on maintenance treatment for severe asthmatics regardless of the phenotype (136). Surprisingly, the current available evidence of tezepelumab in treating AD is not satisfactory although TSLP plays an important role in the pathogenesis of AD (173). Since overwhelming basic studies have demonstrated TSLP is a hopeful target against AD, new agents targeting TSLP or TSLPR are still given great expectations. Encouragingly, Adhikary and coworkers have developed a first potent and safe small-molecule TSLP inhibitor (BP79) leveraging an organ-on-chip technology (195). Application of BP79 onto atopic skin models co-cultivated with lung models has demonstrated its potential to effectively suppress immune cell infiltration and cytokine/chemokine secretion, while also upregulating skin barrier proteins (195). These effects suggest that BP79 may be a promising therapeutic option for the treatment of AD, and has a great potential to expand the therapeutic options in other atopic diseases such as asthma (195). The efficiency targeting IL-33 (itepekimab) and its receptor (astegolimab) for asthma have been confirmed in clinical trials (138), while they are failed to reach statistical significance in AD except etokimab (a humanized anti-IL-33 monoclonal antibody). However, there are lack of research of etokimab in treating asthma (169). The promising efficiency of SM17 targeting IL-25 has been reported in pre-clinical studies both in asthma and AD (143, 144), which could be a hopeful biologic for asthma and AD, even asthma combined AD.

**Table 2:** Biological therapies for atopic dermatitis and asthma.

Biotherapy	Mepolizumab		Dupilumab		Tezepelumab		Itepekimab		Astegolimab		SM17		BP79	
Disease	Asthma	AD	Asthma	AD	Asthma	AD	Asthma	AD	Asthma	AD	Asthma	AD	Asthma	AD
Date of first approval	2015-11-04	/	2018-10-19	2017-03-28	2021-12-17	/	/		/		/		/	
Highest R&D stage	Approved listing	Clinical Phase 2	Approved listing		Approved listing	Clinical Phase 2a	Clinical Phase 3		Clinical Phase 3		Clinical Phase 1		Atopic skin models	
Target	IL-5		IL-4/IL-13Rα		TSLP		IL-33		IL-33R		IL-25		TSLP	
Molecular mechanism	Blocking the binding of IL-5 to IL-5Rα		Dual-receptor antagonism of IL-4/IL-13		Blocking TSLP from binding to its receptor complex		Blocking the binding of IL-33 to IL-33Rα		Inhibits the IL-33 receptor, ST2		Inhibit IL-25-driven ERK1/2 and STAT5 activation		Disrupting TSLP-TSLPR interactions and subsequently blocking downstream signaling events	
Efficiency	Reduced exacerbations, reduced symptoms, small or moderate effect on FEV_1_; reduction or withdrawal of OGs if blood eosinophils >150/μl; improved quality of life.	Mepolizumab did not show clinical efficacy.	Reduced exacerbations, re duced symptoms, improved lung function; decrease or withdrawal of OGs, irrespective of blood eosinophil count at baseline; improved quality of life.	Dupilumab significantly reduced the number of ILC2s in peripheral blood and total serum IgE, significantly improved the patient's dermatitis.	Reduced exacerbations, reduced symptoms, improved lung function; improved quality of life.	Tezepelumab plus TCS tended to have a better EASI75 response.	Itepekimab led to a lower incidence of events indicating a loss of asthma control and improved lung function.	Itepekimab did not show a significant difference in meaningful improvements in EASI scores.	Astegolimab reduced the annualized AER in a broad population of patients.	Astegolimab did not show a significant difference in meaningful improvements in EASI scores.	SM17 could significantly suppress T2 inflammation in the BALF ,effectively reduced goblet cell hyperplasia in the bronchial epithelium and the infiltration of mast cells and eosinophils into the lungs.	SM17 could suppress T2 inflammatory response, reduced Th2 cytokine levels and epidermal thickness, as well as the infiltrations of mast cells, Th2 cells and eosinophils into the dorsal skin and ear tissues.	/	BP79 effectively suppressed immune cell infiltration and IL-13, IL-4, TSLP, and periostin secretion, while upregulating skin barrier proteins.
Effect on ILC2s	Reduced ILC2s proliferative capacity and expression of TSLPR, GATA3 and NFATc1.	/	Reduced ILC2s numbers in the blood of asthmatics and inhibited the expression of IL-5 and IL-13 mRNA in ILC2s.	The absolute numbers and the percentage of Th2 cells (CD4+, CCR6−, CXCR3−, and CCR4+) and ILC2s (Lin−, CD127^+^, and CRTH2^+^) were significantly decreased.	The effect of tezepelumab on these biomarker levels may be related to decreased IL-5 and IL-13 levels.	Tezepelumab interacts with TSLP at the level of its binding site for TSLPR, thus preventing the access of TSLP to its receptor and reduced ILC2s proliferative capacity.	Prevent the activation of ILC2s cells by blocking the action of IL-33.	/	Indirectly regulates the activity and function of ILC2s by inhibiting the IL-33/ST2 signaling pathway and affecting the microenvironment of ILC2s.	/	SM17 binds to IL-17RB on ILC2s and inhibits IL-25-mediated anaphylaxis, thereby reducing ILC2s proliferation.	Inhibit IL-25- driven ERK1/2 and STAT5 activation for achieving anti- Th2 effect and inhibited ILC2s expansion and Th2 cytokine Release.	/	Abrogated TSLP-mediated proliferation of CD4^+^ T cells, reduced the expression of TSLP, IL-5, IL-2, IL-4, IL-9, IL-13, IFNγ, IL-22, and TNFα.
Reference	126	194	130	166	137	172	138	169	139	169	144	143	/	195

ILC2s, group 2 innate lymphoid cells; T2, type 2; Th2, type 2 helper T; IL, Interleukin; IL-13Rα, Interleukin-13 receptor α; IL-33R, Interleukin-33 receptor; TSLP, Thymic stromal lymphopoietin; ST2, serum stimulating factor-2; OGs, oral glucocorticoids; EASI, eczema area and severity index; AER, asthma exacerbation rate; FEV1, forced expiratory volume in 1 second; BALF, bronchoalveolar lavage fluid; TCS, topical corticosteroids.

## 6 Concluding remarks

As an important treatable treat of asthma, AD is not only trigger but also exacerbates asthma. This review suggests that ILC2s bridge the pathophysiological mechanisms of asthma combined with AD, and making them a promising target. However, to date, despite the availability of dupilumab for the treatment of asthma combined with AD, further research is still needed for other biologics/therapies targeting ILC2s. Particularly, new agents targeting TSLP or TSLPR such as ASP7266 (a novel recombinant monoclonal antibody against TSLPR) and BP79 (a small- molecular inhibitor against TSLP-TSLPR), which crucial role is demonstrated by overwhelming basic studies are given great expectations in treating asthma combined with AD, although there is no clinical trial supporting the efficacy of TSLP-targeting drugs against AD at present. With the advancement of technology, more and more new technologies, such as organs-on-chips, are facilitating rapid evaluation of new drug development. Undoubtedly, biologic agents serve as highly effective supplementary therapies for severe, refractory asthma and AD. Given their high cost, it is imperative to target their use specifically to patients who are most likely to derive significant benefit from these antibodies.

Furthermore, agents that regulate the functions of ILC2s continue to be anticipated, including activators or inhibitors of PD-1/PD-L1 or PD-L2, SCFAs, dopamine, hormones, and other entities. Fascinatingly, a recent study has unveiled the immunomodulatory function of the Ca2^+^ release-activated Ca2^+^ channel components Orai1 and Orai2 in mouse ILC2s (203). Blocking or genetic ablation of Orai1 and Orai2 downregulated mouse ILC2s effector functions and cytokine productions, and attenuated ILC2-mediated airway inflammation and AHR (203). This discovery holds promise for the development of novel therapies aimed at improving asthma and AD, paving the way for potential downstream targets in the treatment of asthma comorbid with AD.
